# Hemophagocytic Lymphohistiocytosis Induced Cytokine Storm Secondary to Human Immunodeficiency Virus Associated Miliary Tuberculosis

**DOI:** 10.7759/cureus.6589

**Published:** 2020-01-07

**Authors:** Meghana Parsi, Kinjal Dargan

**Affiliations:** 1 Internal Medicine, Crozer-Chester Medical Center, Upland, USA; 2 Hematology/Oncology, Cooper University Medical Center, Camden, USA

**Keywords:** hemophagocytic lymphohistiocytosis, hlh, hiv, aids, mycobacterium tuberculosis, tb, tb-hlh

## Abstract

Hemophagocytic lymphohistiocytosis (HLH) is a rarely diagnosed fatal inflammatory disease associated with an overactive immune system. It occurs in a host of conditions, with human immunodeficiency virus (HIV) being a rare but serious cause, usually occurring in patients with acquired immunodeficiency syndrome (AIDS). The diagnosis of HLH can be very difficult, as it presents with vague signs and symptoms, which can be present in multiple diseases. This case highlights the diagnostic dilemma faced when treating this potentially fatal condition. Usually, treating the underlying trigger for HLH is sufficient to counteract the overwhelming inflammatory response; however, this can prove to be difficult, as demonstrated in our patient. We present a case of miliary tuberculosis in the setting of HIV/AIDS, complicated by HLH in a young male patient. Whether it was due to delays in treatment or the rapidly fulminant nature of the disease, our patient had a poor clinical outcome. Although rare, tuberculosis-associated HLH must be considered as a cause of secondary HLH in all patients, especially those who are immunosuppressed.

## Introduction

Hemophagocytic lymphohistiocytosis (HLH) is a rare inflammatory condition associated with an intense cytokine release and robust immune activation (histiocytes and lymphocytes). Undiagnosed and untreated cases of HLH have a high mortality rate. Various forms of HLH exist, with the hereditary form present in children as young as 18 months. A secondary form of HLH is commonly associated with a variety of infections, malignancies, autoimmune conditions, organ transplantation, and immunosuppressive states [1]. Human immunodeficiency virus (HIV) with its host of opportunistic infections is a common immunodeficiency syndrome that triggers HLH. Mycobacterium tuberculosis (TB) associated HLH (TB-HLH) in a patient with HIV is a rare and underdiagnosed entity. While primary TB infection is asymptomatic in a majority of the cases, it can rarely disseminate and lead to severe disease. Nonspecific and vague symptoms such as fatigue, fever, and weight loss are common to many conditions including HLH, often making its diagnosis very difficult [2]. This emphasizes the importance of strong clinical suspicion and prompt detection of HLH, as delays in treatment can prove to be fatal. We describe here a case of HLH secondary to miliary TB in a patient with advanced acquired immunodeficiency syndrome (AIDS).

## Case presentation

A 28-year-old African-American male with no significant medical history presented with a one-month history of 20-lb unintentional weight loss, drenching night sweats, subjective fevers, malaise, and decreased appetite. He was not an intravenous drug user but reported multiple sexual encounters with both males and females. He presented acutely ill, cachectic, and lethargic in appearance. Vital signs revealed a temperature of 101.1F, heart rate was tachycardic but regular at 143 beats per minute, respiratory rate of 22 cycles per minute, and SpO2 of 97% on room air. Laboratory studies are provided in Table 1.

**Table 1:** Significant labs WBC, white blood cells; HIV, human immunodeficiency virus; AST, aspartate aminotransferase; ALT, alanine aminotransferase; LDH, lactate dehydrogenase; IL-2, interleukin-2

Parameter (Normal Range)	Admission	Day 10	Day 15
Hemoglobin (14-8 g/dL)	8.9	6.1	7.8
WBC (4,500-11,000 cells/µL)	3,660 cells/µL	1,220 cells/µL	5,460 cells/µL
Platelets (150-400×10^3^/µL)	66×10^3^/µL	38×10^3^/µL	68×10^3^/µL
Creatinine (0.6-1.2 mg/dL)	1.0	1.2	2.4
HIV antigen/antibody	Positive		
CD4 count (360-1,725/mm^3^)	149		
HIV viral load	2.5×10^9 ^copies/mL		
Total bilirubin (0.1-1.2 mg/dL)	1.4	2.3	
AST (13-40 U/L)	307	1,787	
ALT (10-59 U/L)	74	305	
Alkaline phosphatase (39-117 U/L)	295	367	
Lactic acid (4.5-8.0 mg/dL)	2.7	4.4	
LDH (<37 U/L)		8,400	
Uric acid (3.5-8.5 mg/dL)		11.9	
Triglycerides (<150 mg/dL)	595	581	696
Ferritin (15-400 ng/mL)		>100,000	
CD25 (soluble IL-2 receptor) (≥2,400 U/mL)		>2,500	
Fibrinogen (163-398 mg/dL)		102	

Testing for HIV was positive with a CD4 count of 149/mm^3^ (reference range: 360-1,725/mm^3^) and a viral load of 2.5x10^9^. A computed tomography (CT) of the chest showed diffuse miliary nodules, a trace right pleural effusion, and bilateral mediastinal and supraclavicular lymphadenopathy. A CT of the abdomen and pelvis revealed hepatosplenomegaly and mesenteric edema. In the setting of febrile neutropenia, the patient was started on broad-spectrum antibiotics and trimethoprim-sulfamethoxazole for Pneumocystis jiroveci pneumonia prophylaxis. Subsequent BAL (bronchoalveolar lavage), AFB (acid-fast bacillus) sputum, and urine culture were positive for Mycobacterium TB seven days into admission. Ten days into hospital admission, he developed hypoxic respiratory failure with increasing oxygen requirement. Repeat testing revealed worsening of the right pleural effusion and of the miliary nodules. He continued to have persistent low-grade fevers despite being on several broad-spectrum antibiotics, and rifampin, isoniazid, pyrazinamide, and ethambutol (RIPE) for TB. He subsequently developed acute renal failure and septic shock. An extensive search for infection in the pleural fluid, blood, sputum, and urine was nonrevealing. Serological testing for immunoglobulin M/immunoglobulin G (IgM/IgG) Epstein-Barr virus, IgM/IgG cytomegalovirus, aspergillosis, histoplasmosis, toxoplasmosis, and herpes simplex virus were negative. His liver function tests were worsening, as illustrated in Table 1. An ultrasound of the abdomen demonstrated marked hepatosplenomegaly and a patent portal vein with no evidence of biliary obstruction. Testing for hepatitis A, hepatitis B, and hepatitis C viruses was negative. An extensive rheumatological workup was negative for antinuclear antibodies and antineutrophil cytoplasmic antibodies.

**Figure 1:**
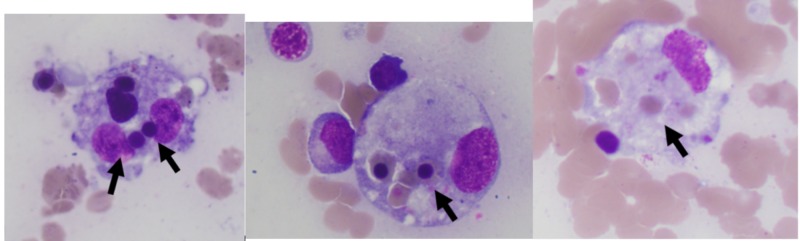
3 Medium power stains of bone marrow smears Foamy macrophages are seen engulfing precursor erythrocytes (arrows)

Eight days into hospital admission, he had worsening pancytopenia. Peripheral smear revealed thrombocytopenia but was negative for schistocytes. While hemolysis testing was negative, labs revealed an elevated uric acid, lactate dehydrogenase, and elevated ferritin, which was concerning for an underlying malignancy. The persistent fevers, splenomegaly, and worsening pancytopenia prompted a bone marrow biopsy, which was performed on day 10 of admission. This demonstrated a hypercellular bone marrow (>95%) with severe hemophagocytosis, without evidence of an underlying fungal, neoplastic, or malignant process (Figure 1).

Tandem flow cytometry revealed a severely reversed CD4/CD8 ratio. Fever, splenomegaly, pancytopenia, and further lab testing revealing hyperferritinemia, elevated CD25, hypofibrinogenemia, and hemophagocytosis (in the bone marrow) fulfilled and confirmed the diagnosis of HLH. A diagnosis of secondary HLH in the setting of AIDS-associated miliary TB was made. Due to the fear of triggering an immune reconstitution syndrome (IRIS), he was not initiated on highly active antiretroviral therapy (HAART) therapy with bictegravir/emtricitabine/tenofovir until day 30 of admission. HLH-2004 regimen, which includes treatment with the immunosuppressive etoposide and the anti-inflammatory dexamethasone, was offered. The patient opted against chemotherapy but agreed to two weeks of dexamethasone at a dose of 10 mg/m^2^, which was started on day 10 of admission. There was a slight improvement in his counts, but his hospital course was soon complicated by a gastrointestinal bleed, abdominal perforation, and ventilator-dependent respiratory failure, after which his family decided to pursue comfort care. He passed away shortly thereafter.

## Discussion

HLH is a life-threatening aggressive form of immune system overactivation. It is characterized by the dysregulation of cytotoxic T cell and natural killer (NK) cell function, ultimately leading to a cytokine burst and the proliferation of lymphocytes and histiocytes. These activated cells can proliferate in various organs and lead to multiorgan failure. Hemophagocytosis, the hallmark of HLH, involves the pathological finding of activated histiocytes engulfing erythrocytes, leukocytes, platelets, and their precursor cells [3].

Even with a well-defined diagnostic criterion (Table 2), HLH is difficult to diagnose because of its variable presentation and association with other diseases [4]. Patients with HLH present with a variety of nonspecific clinical features such as fever, fatigue, hepatosplenomegaly, and pancytopenia. In patients with HIV/AIDS, there is a broad range of differentials for fever, fatigue, splenomegaly, and pancytopenia. With overlapping clinical features and rarity of HLH, making a diagnosis of HLH in the setting of HIV/AIDS can be very difficult [5]. Ng et al. reported the case of a patient in which the diagnosis of HLH was delayed for over a month [6]. This was further supported in a postmortem analysis of 107 critically ill patients, where HLH was found in 69 (64%) patients [7]. This indicates that HLH is underdiagnosed and undertreated.

**Table 2:** Diagnostic criteria for HLH The diagnosis of HLH requires the presence of at least five of the eight mentioned indices HLH, hemophagocytic lymphohistiocytosis; IL-2Rα, interleukin-2 receptor α; NK, natural killer

HLH-2004 Protocol
Fever ≥ 38.5°C
Splenomegaly
Peripheral blood cytopenias with at least two of the following: absolute neutrophil count < 1,000/µL, hemoglobin < 9 g/dL, platelets < 100,000/µL
Hypertriglyceridemia (>265 mg/dL) or hypofibrinogenemia (<150 mg/dL)
Hemophagocytic lymphocytes in the bone marrow, lymph node, spleen or liver
Elevated soluble CD25 (IL-2Rα chain ) ≥ 2,400 U/mL
Low or absent NK cell activity
Ferritin ≥ 500 ng/mL

Primary or familial HLH is thought to be due to mutations in the genes responsible for cytokine release. However, the mechanism behind secondary HLH (AKA macrophage activation syndrome), which occurs in the setting of various infectious and inflammatory conditions, is unclear [8]. HIV infection can be associated with HLH in several different ways. In the majority of cases, it is described in patients with long-standing AIDS and associated opportunistic infection. It has also been described during the acute seroconversion stage of HIV as well as in the setting of IRIS within weeks of initiation of HAART [9-10]. With the initiation of antiretroviral therapy, there can be unmasking of an infection or the worsening of a previously controlled opportunistic infection. Surprisingly, even patients with well-controlled HIV, with undetectable viral loads, can develop HLH, proving that it is not limited to those who are overwhelmingly immunosuppressed [11]. An autopsy study of 56 patients with AIDS found an approximate 20% prevalence of hemophagocytosis, suggesting the possibility of a subclinical form of HLH [12].

Our patient had several independent risk factors for HLH, including untreated HIV and disseminated miliary TB. As we know, HLH is caused by alterations in cytotoxic T cell and NK cell function, leading to uncontrolled cytokine production, subsequent phagocytosis, and multiorgan failure. Numerous studies propose that HIV is associated with higher than normal levels of IL-10 (interleukin-10), tumor necrosis factor (TNF)-alpha, and interferon (IFN)-gamma. This cytokine dysregulation is thought to occur within seven days of detectable HIV viremia. Similarly, levels of TNF-alpha and IFN-gamma are thought to be elevated in patients with TB as well. After phagocytosis, Mycobacterium TB can act as an intracellular obligate organism to induce a Th1 immune response and further propagate cytokine production and immune destruction [13].

The most important and immediate goal of therapy is to control the underlying cytokine storm and dysregulated immune proliferation. Conventional therapy for HLH is based on recommendations by Histiocyte Society HLH-2004, which includes induction chemotherapy with etoposide, cyclosporine, and dexamethasone for eight weeks, and subsequent bone marrow transplant for familial or refractory cases [1]. However, this may be avoided in stable patients. In such cases, treating the underlying infection or trigger, along with steroids (to offset the extensive inflammation), may be sufficient. Untreated HLH has a mortality of 100%, but timely initiation of anti-tubercular therapy (ATT) can reduce the mortality rate by 40-50% and timely initiation of HAART can reduce mortality by 70% [13].

In contrast to the later stages of HIV infection, the prognosis of HLH in patients with acute HIV infection seems to be more favorable. A study of nine patients with HLH and acute HIV infection achieved complete recovery with the administration of HAART, intravenous immunoglobulins, or steroids. In a study evaluating 28 patients with HLH in the setting of advanced AIDS with opportunistic infections, there was a recovery rate of only 21% (6 out of 28) [14-16]. This proves the need for prompt and early initiation of HAART for all patients once diagnosed with HIV before the development of full-blown AIDS. HAART was not started until one month into admission in our patient. It is unclear whether early initiation of treatment would have made a difference in his overall condition.

A review of the literature has revealed only 79 other cases of TB-HLH [17-19]. Padhi et al. reviewed 63 cases of these cases, which have been described from 1980 to 2014. Out of these cases, 65% of the cases had another co-morbidity, with only three of them having HIV/AIDS. Out of these, ATT was initiated in 86% (54/63) of patients, with a mortality rate of 49% (31/63) [20]. In most cases, the high fatality rate was attributed to delayed diagnosis and initiation of treatment. A majority of these patients were critically ill and had underlying sepsis, complicating and confounding the clinical course.

While studies have shown good results, better prognosis, and increased survival with the use of etoposide, it has yet to be implemented on a widespread basis. Data regarding the safety, efficacy, and use of such a chemotherapeutic regimen in patients with severe sepsis, multiorgan failure, or severe immunosuppression, such as our patient, are lacking. The use of chemotherapy will undoubtedly increase the burden of illness in the critically ill. In such cases, immunosuppression with high dose steroids remains crucial in controlling systemic inflammation. Further recommendations regarding which patients require the complete HLH-2004 protocol need to be made. For now, targeting and treating the underlying disease trigger is critical. Our patient was started on ATT on day 7 of admission and high-dose steroids on day 10 of admission, but he did not receive HAART therapy until day 30 of admission. Unfortunately, our patient had a rapid and deteriorating course of disease progression, making it hard to determine if he would have recovered.

## Conclusions

In all immunosuppressed patients, the possibility of HLH should be kept in mind in the differential for any patient with persistent fever, cytopenias, and splenomegaly. Especially in patients with a known diagnosis of HIV, one must consider opportunistic infections such as TB as a secondary cause of HLH. We advise that treatment or even prophylaxis be considered in all high-risk patients. The use of the HLH-2004 protocol in patients with HLH and underlying HIV/AIDS is currently controversial. Further studies are necessary to help guide the use of such a regimen in these high-risk patients.

## References

[1] Henter JI, Aricò M, Egeler RM (1997). HLH-94: a treatment protocol for hemophagocytic lymphohistiocytosis. HLH Study Group of the Histiocyte Society. Med Pediatr Oncol.

[2] Garcia C, Haye K, Gabig T (2016). Hemophagocytic lymphohistiocytosis (HLH): a case series and review. Am J Med Case Rep.

[3] Rosado FG, Kim AS (2013). Hemophagocytic lymphohistiocytosis: an update on diagnosis and pathogenesis. Am J Clin Pathol.

[4] Henter JI, Horne A, Aricó M (2007). HLH-2004: Diagnostic and therapeutic guidelines for hemophagocytic lymphohistiocytosis. Pediatr Blood Cancer.

[5] Kim SB, Shrivastava MS, Strakhan M (2015). A rare cause of acquired immune deficiency syndrome related pancytopenia. Hematol Rep.

[6] Ng D, Ghosh N, Hicks LK (2020). Hemophagocytic lymphohistiocytosis—late diagnosis in an adult patient. BMJ Case Rep.

[7] Strauss R, Neureiter D, Westenburger B (2004). Multifactorial risk analysis of bone marrow histiocytic hyperplasia with hemophagocytosis in critically ill medical patients—a postmortem clinicopathologic analysis. Crit Care Med.

[8] Verbsky J. W., Grossman W. J (2006). Hemophagocytic lymphohistiocytosis: diagnosis, pathophysiology, treatment, and future perspectives. Ann Med.

[9] Sun HY, Chen MY, Fang CT (2004). Hemophagocytic lymphohistiocytosis: an unusual initial presentation of acute HIV infection. J Acquir Immune Defic Syndr.

[10] Huang DB, Wu JJ, Hamill RJ (2004). Reactive hemophagocytosis associated with the initiation of highly active antiretroviral therapy (HAART) in a patient with AIDS. Scand J Infect Dis.

[11] Thoden J, Rieg S, Venhoff N (2012). Fatal hemophagocytic syndrome in a patient with a previously well-controlled asymptomatic HIV infection after EBV reactivation. J Infect.

[12] Niedt GW, Schinella RA (1985). Acquired immunodeficiency syndrome. Clinicopathologic study of 56 autopsies. Arch Pathol Lab Med.

[13] Jung YJ, Ryan L, LaCourse R, North RJ (2005). Properties and protective value of the secondary versus primary T helper type 1 response to airborne Mycobacterium tuberculosis infection in mice. J Exp Med.

[14] Bhatia S, Bauer F, Bilgrami SA (2003). Candidiasis-associated hemophagocytic lymphohistiocytosis in a patient infected with human immunodeficiency virus. Clin Infec Dis.

[15] Chen TL, Wong WW, Chiou TJ (2003). Hemophagocytic syndrome: an unusual manifestation of acute human immunodeficiency virus infection. Int J Hematol.

[16] Pellegrin JL, Merlio JP, Lacoste D, Barbeau P, Brossard G, Beylot J, Leng B (1992). Syndrome of macrophagic activation with hemophagocytosis in human immunodeficiency virus infection. Rev Med Interne.

[17] Yun Z, Guangyu L, Hongli Q (2017). Tuberculosis-associated hemophagocytic lymphohistiocytosis with initial presentation of fever of unknown origin in a general hospital: an analysis of 8 clinical cases. Medicine.

[18] Ray U, Dutta S, Bandyopadhyay S, Mondal S (2019). Infections and HLH - experience from a tertiary care centre. J Assoc Physicians India.

[19] Brastianos PK, Swanson JW, Torbenson M, Sperati J, Karakousis PC (2006). Tuberculosis-associated hemophagocytic syndrome. Lancet Infect Dis.

[20] Padhi S, Ravichandran K, Sahoo J, Varghese RG, Basheer A (2015). Hemophagocytic lymphohistiocytosis: an unusual complication in disseminated Mycobacterium tuberculosis. Lung India.

